# Disrupted gray matter connectome in vestibular migraine: a combined machine learning and individual-level morphological brain network analysis

**DOI:** 10.1186/s10194-024-01861-9

**Published:** 2024-10-11

**Authors:** Wen Chen, Hongru Zhao, Qifang Feng, Xing Xiong, Jun Ke, Lingling Dai, Chunhong Hu

**Affiliations:** 1 Department of Radiology, The First Affiliated Hospital of Soochow University, Shizi Street 188, Suzhou, Jiangsu 215006 P.R. China; 2 Institute of Medical imaging, Soochow University, Soochow, Jiangsu Province People’s Republic of China; 3 Department of Neurology, The First Affiliated Hospital of Soochow University, Suzhou, Jiangsu 215006 China

**Keywords:** Vestibular migraine, Magnetic resonance imaging, Gray matter, Morphological brain network, Graph theory, Machine learning

## Abstract

**Background:**

Although gray matter (GM) volume alterations have been extensively documented in previous voxel-based morphometry studies on vestibular migraine (VM), little is known about the impact of this disease on the topological organization of GM morphological networks. This study investigated the altered network patterns of the GM connectome in patients with VM.

**Methods:**

In this study, 55 patients with VM and 57 healthy controls (HCs) underwent structural T1-weighted MRI. GM morphological networks were constructed by estimating interregional similarity in the distributions of regional GM volume based on the Kullback–Leibler divergence measure. Graph-theoretical metrics and interregional morphological connectivity were computed and compared between the two groups. Partial correlation analyses were performed between significant GM connectome features and clinical parameters. Logistic regression (LR), support vector machine (SVM), and random forest (RF) classifiers were used to examine the performance of significant GM connectome features in distinguishing patients with VM from HCs.

**Results:**

Compared with HCs, patients with VM exhibited increased clustering coefficient and local efficiency, as well as reduced nodal degree and nodal efficiency in the left superior temporal gyrus (STG). Furthermore, we identified one connected component with decreased morphological connectivity strength, and the involved regions were mainly located in the STG, temporal pole, prefrontal cortex, supplementary motor area, cingulum, fusiform gyrus, and cerebellum. In the VM group, several connections in the identified connected component were correlated with clinical measures (i.e., symptoms and emotional scales); however, these correlations did not survive multiple comparison corrections. A combination of significant graph- and connectivity-based features allowed single-subject classification of VM *versus* HC with significant accuracy of 77.68%, 77.68%, and 72.32% for the LR, SVM, and RF models, respectively.

**Conclusion:**

Patients with VM had aberrant GM connectomes in terms of topological properties and network connections, reflecting potential dizziness, pain, and emotional dysfunctions. The identified features could serve as individualized neuroimaging markers of VM.

**Supplementary Information:**

The online version contains supplementary material available at 10.1186/s10194-024-01861-9.

## Introduction

Vestibular migraine (VM) is a disabling neurological disease characterized by headache and recurrent episodes of vertigo, along with nausea, vomiting, and balance problems [1]. The disease has a lifetime prevalence of 1–3% in the general population [2, 3], and it is recognized as the most common cause of central episodic vertigo, and the second leading etiology of vestibular syndromes [4, 5]. Recurrent vestibular and migraine symptoms severely affect patients’ quality of life, and pose a substantial economic burden on the family and society [3]. However, the current diagnosis of VM mainly relies on medical history and clinical symptoms, with no specific or objective evidence, such as laboratory, imaging, and pathological findings [6, 7]. The unclear pathogenesis of VM is a primary obstacle to achieving optimal diagnosis and clinical efficacy [8]. Therefore, exploring the neural mechanism underlying VM and identifying potential biomarkers are important for facilitating the development of better diagnostic and treatment options for affected patients.

Recently, the use of advanced neuroimaging techniques has greatly broadened our insight into the neural mechanisms of VM. Notably, several studies using resting-state functional MRI (rs-fMRI) have proposed VM as a central vestibular disorder involving complex neuronal networks. For instance, several investigations have shown aberrant functional connectivity of regions regarding pain, vestibular, visual, and emotional processing in patients with VM [9–14]. Other studies using independent component analysis have demonstrated that patients suffering from VM had interruptions in multiple intrinsic neural networks, including sensorimotor, vestibular cortical, visual, salience, executive control, and default mode networks [8, 15–17]. However, the structural basis of the observed abnormalities in functional networks should be elucidated.

To date, structural MRI (sMRI)-based neuroimaging studies on VM have mainly explored regional brain abnormalities in gray matter (GM) volume using a voxel-based morphometry (VBM) approach; however, the results are highly heterogeneous and conflicting [14, 18–21]. This discrepancy may be partially attributed to the instability of conventional univariate (i.e., GM volume) findings, whereas multivariate approaches may better capture complex pathological changes [22]. Furthermore, only regional structural changes could be identified based on VBM, which overlooked the potentially altered interrelationships among brain regions. The human brain is increasingly recognized as a system of interacting information-sharing networks [23]; thus, comprehensive characterization from a network perspective can deepen the understanding of the structural architecture at the whole-brain level in both physical and pathological states [24, 25]. In view of this, structural covariance network (SCN), a type of network constructed based on statistical correlations of the morphological indices among brain regions reflected by sMRI [26], may be a promising tool to provide complementary and comprehensive insight into the neural basis of VM. One strength of SCN lies in the distinct advantages offered by sMRI, including easy acquisition, high signal-to-noise ratio, and relative insensitivity to artifacts [27]. Moreover, several pieces of evidence have indicated that the topological characteristics of the GM network connectome are sensitive to early brain damage [28, 29], thus may provide noninvasive early-stage in vivo biomarkers to assist in the clinical diagnosis of VM.

However, identifying biomarkers based on SCN has encountered certain technical challenges. The major concern arises from the fact that SCNs are usually calculated by constructing a single brain network for each group, which disregards interindividual variability and precludes the examination of brain–behavior relationships and health–disease classification [30]. To address this issue, Kong et al. proposed a novel method that uses the Kullback–Leibler (KL) divergence-based similarity (KLS) measure to evaluate interregional morphological connectivity, enabling the construction of individual GM morphological networks [31]. This methodology effectively overcomes the aforementioned limitations by quantifying morphological relationships in individual subjects, and has been successfully used to delineate alterations in the morphological connectivity profiles of neurological diseases, such as Parkinson’s disease [27, 32], mild cognitive impairment [33], and social anxiety disorder [34]. Combined with the observed neuroimaging findings of GM volume alterations, we hypothesized that patients with VM might exhibit aberrant GM network connectomes that could be detected by KLS-based SCN. This approach may improve our understanding of the underlying structure network mechanisms of VM.

**Fig. 1:**
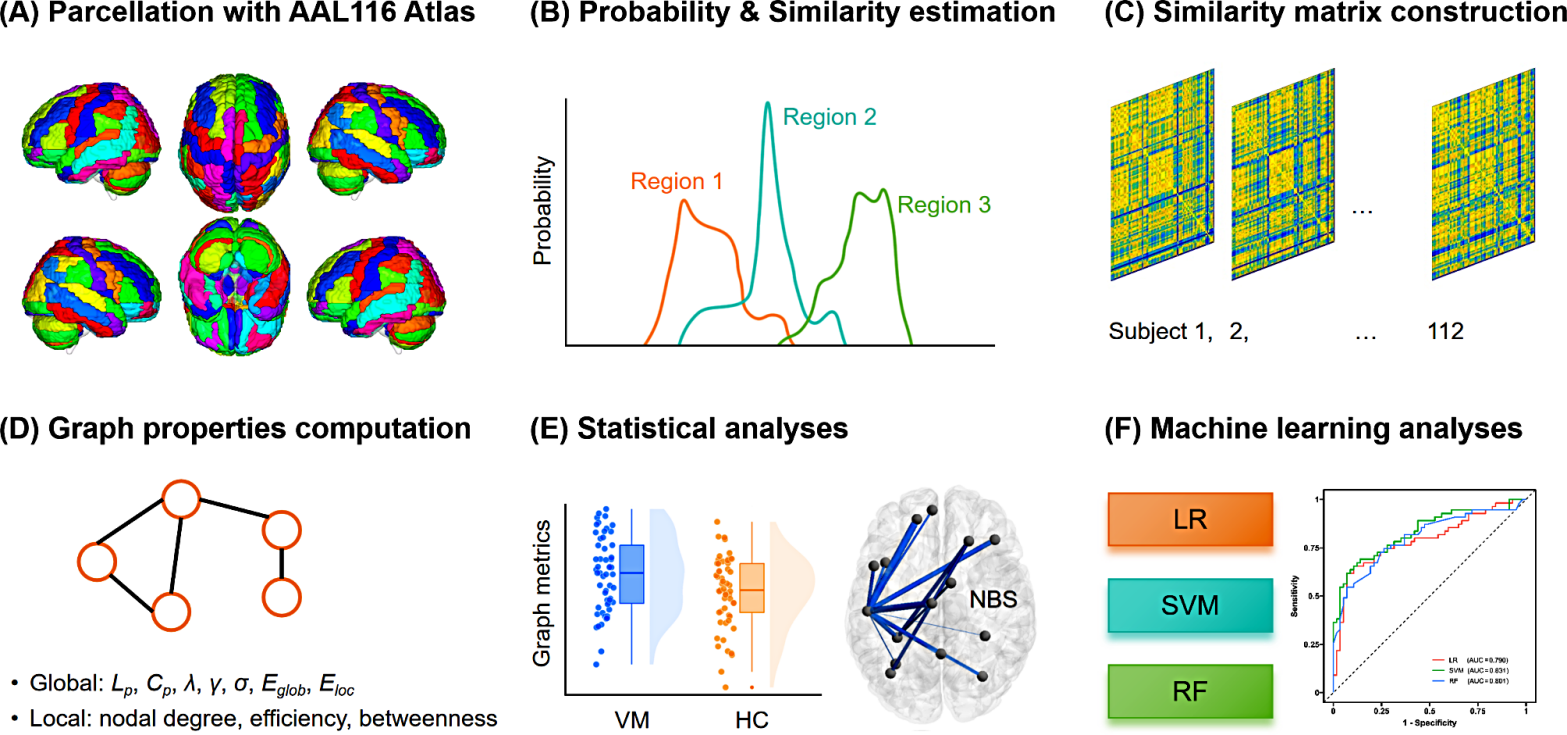
Flowchart of the analysis process. (**A**) The GM volume was estimated with VBM using SPM. Whole-brain GM was parcellated using the AAL116 atlas (116 regions). (**B**) The PDF of GM volume was calculated using kernel density estimation. Statistical similarities between the PDFs of pairwise brain regions were then evaluated with KL divergence. (**C**) As a result, a 116 × 116 similarity matrix representing the GM volume covariance network was generated for each subject. (**D**) Global and local topological properties were calculated through graph-theoretical analyses. (**E**) The network metrics, including network topological properties and connections were compared between patients with VM and HCs. (**F**) LR, SVM, and RF classifiers were used to construct models for distinguishing individuals with VM from HCs based on the discriminative GM connectome features. GM = gray matter; VBM = voxel-based morphometry; SPM = Statistical Parametric Mapping; AAL = Automatic Anatomical Labeling; PDF = Probability Distribution Function; KL = Kullback–Leibler; VM = vestibular migraine; HC = healthy control; LR = logistic regression; SVM = support vector machine; RF = random forest; *L*_*p*_ = characteristic path length; *C*_*p*_ = clustering coefficient; *λ* = normalized characteristic path length; *γ* = normalized clustering coefficient; *σ* = small-worldness; *E*_*glob*_ = global efficiency; *E*_*loc*_ = local efficiency; NBS = network-based statistics; AUC = area under the curve

Therefore, the purpose of this study was to verify our hypothesis by investigating the topological organization of GM morphological networks in patients with VM. In particular, both graph-theoretical metrics and interregional morphological connectivity were computed to systematically estimate differences in network attributes between patients with VM and healthy controls (HCs). Furthermore, because machine learning has received increasing attention and significantly contributes to the identification of potential neuroimaging biomarkers [35], we used three common machine learning models, i.e., logistic regression (LR), support vector machine (SVM), and random forest (RF), to test whether GM connectome features can be used to differentiate patients with VM from HCs (Fig. 1).

## Materials and methods

### Subjects and clinical assessment

This study was conducted according to the Declaration of Helsinki and was approved by the Ethics Committee of the First Affiliated Hospital of Soochow University. Written informed consent was obtained from all subjects before their participation. Fifty-five patients with VM (47 females/8 males; age: 45.89 ± 12.29 years old; education level: 10.47 ± 4.79 years) and 57 demographically similar healthy controls (HCs) (48 females/9 males; age: 45.86 ± 12.37 years old; education level: 11.89 ± 4.77 years) were included in this study. The data presented here derived from our dataset collected between October 2020 and December 2022, and was partially overlapped with that employed in our previous publication on functional concordance [36]. In this study, the level of demographic factors between groups were matched to be as close as possible under the condition of sufficient and balanced sample sizes [37], and T1 structural images were utilized as the sole and principal image data. The diagnosis of VM were based on the criteria published by the Bárány Society and International Headache Society (ICHD-3 beta, appendix) [38, 39]. Videonystagmography, vestibular caloric test, video head impulse and audiometry tests were performed to rule out peripheral vestibular diseases. We collected demographic and clinical data from all patients using a standardized questionnaire, including sex, age, education level, migraine disease duration, vertigo disease duration, headache frequency (days per month), 10-point Visual Analog Scale (VAS), Dizziness Handicap Inventory (DHI), Migraine Disability Assessment Scale (MIDAS), Headache Impact Test-6 (HIT-6), Patient Health Questionnaire-9 (PHQ-9), and Generalized Anxiety Disorder-7 (GAD-7).

All patients with VM were not under regular preventive therapy and had not taken any therapeutic drugs within 3 days before MRI scanning. We performed MRI for patients with VM during the interictal stage. Patients were considered to be in the interictal period if they did not have vestibular or migraine symptoms at least 3 days before and 1 day after MRI acquisition. All the HCs had no history of migraine, vertigo, or any other types of primary headache. The following exclusion criteria were applied to all subjects: left-handedness; other neurological or psychiatric diseases; other pain conditions; drug or alcohol abuse; and MRI contraindications, such as pregnancy, claustrophobia, and ferromagnetic implants.

To determine whether the sample size was adequate in the current study, a power analysis was further conducted. Since there are no studies directly exploring the GM connectome in patients with VM, we reviewed previous literature of structural neuroimaging studies on VM (with a case-control design), and listed the effect sizes in Table S1. The minimum Cohen’s d in each research ranged from 1.02 to 1.47, which indicated extremely large effects, and we finally applied a conservative Cohen’s d of 0.80 (corresponding to a large effect). The G*Power software [40] was then used to estimate the required sample size with the following parameters: test family = t tests, statistical test = difference between two independent groups, tail(s) = two, effect size d = 0.80, significance criterion α = 0.05, and statistical power 1-β = 0.8. The required sample size was determined to be 26 for each group, indicating that our sample of 55 patients with VM and 57 HCs was adequate to provide sufficient statistical power for analyses.

### MRI acquisition

All subjects were scanned using a 3.0-Tesla MRI system (MAGNETOM Skyra, Siemens Healthcare, Erlangen, Germany) with a 16-channel head and neck joint coil. Head motion and scanning noise were reduced by applying foam padding and earplugs. All subjects were instructed to lie still in the supine position with their eyes closed, relaxing and staying awake. High-resolution T1-weighted anatomic images were obtained using a sagittal fast spoiled gradient-recalled echo sequence: repetition time = 2300 ms, echo time = 2.98 ms, field of view = 256 × 256 mm^2^, matrix = 256 × 256, slice thickness = 1 mm, and slice number = 192. After the procedure, the images were immediately reviewed by two experienced radiologists to rule out visible lesions.

### Data preprocessing

For structural MRI data processing, we used the Statistical Parametric Mapping analysis package (SPM12, http://www.fil.ion.ucl.ac.uk/spm/software/spm12/) along with the Computational Anatomy Toolbox for SPM (CAT12, http://www.neuro.uni-jena.de/cat/) for VBM analysis. In this procedure, all T1-weighted images were corrected for bias field inhomogeneities and then segmented into GM, white matter, and cerebrospinal fluid. The images were transformed into the standard Montreal Neurological Institute space by normalizing with a transformation integration of the diffeomorphic anatomic registration through exponentiated lie algebra algorithm and geodesic shooting, and resampled to 1.5 × 1.5 × 1.5 mm^3^. To preserve tissue volume after warping, voxel values in individual GM images were modulated by multiplying by Jacobian determinants derived from normalization. Finally, a GM volume map of each subject was created.

### Construction of individual GM morphological networks

Based on the Automatic Anatomical Labeling atlas, the brain was divided into 116 regions of interest (ROIs), which served as nodes of the morphological brain network. Consistent with previous reports [32, 34], KL divergence was used to quantify the similarity of regional GM volume distribution between two regions (i.e., the edge between nodes), referred to as a morphological connection. To construct the GM morphological networks of each subject, we first extracted the GM volume values of all voxels within each ROI. The probability density function of these values was estimated using kernel density estimation with automatically estimated bandwidths, which was in turn used to compute the Probability Distribution Function (PDF) [41]. The KL divergence was then computed between two PDFs of each pair of ROIs. KL divergence quantifies the difference between two probability distributions, which equates to the information lost when a probability distribution is used to approximate another [42]. The standard KL divergence from the distribution *Q* to *P* is computed as follows:

$${D_{KL}}(P\parallel Q)\, = \,\sum\limits_{i = 1}^n {P(i)\log {{P(i)} \over {Q(i)}},}$$

thus a 116 × 116 similarity matrix was generated for each subject by calculating the KLS values between all possible pairs of the 116 ROIs. However, because *D*_KL_(*P*‖*Q*) is unequal to *D*_KL_(*Q*‖*P*), we assessed the similarity between the two PDFs using a symmetric KL divergence, that is, *D*_KL_(*P*, *Q*) [31, 41], which is a derivate of the KL divergence and is calculated as follows:

$${D_{KL}}(P,Q)\, = \,\sum\limits_{i = 1}^n {\left( {P(i)\log {{P(i)} \over {Q(i)}} + Q(i)\log {{Q(i)} \over {P(i)}}} \right)}.$$

Subsequently, the value of symmetric KL divergence was converted to a similarity measurement (range 0 [no similarity] to 1 [identical distributions]) for all pairwise regions using the following formula [31, 41]:

$${\rm{KLS(}}P,Q)\, = \,{e^{ - {D_{{\rm{KL}}}}(P,Q)}},$$

and this produced 1 KLS-based SCN (116 × 116) for each subject, which was recognized as the final GM morphological network.

### Network analyses

Network analyses were performed using the GRETNA toolbox (https://www.nitrc.org/projects/gretna/). To ensure that the morphological networks contained the same number of edges across participants at a fixed sparsity (i.e., the number of actual edges as a fraction of all possible edges), we used a sparsity-based thresholding procedure to convert them to a set of binary networks. A wide range of sparsity thresholds (0.05–0.30, with an interval of 0.01) [34] were used for binarization. In the resulting binarized SCNs, a value of 1 denotes significant covariation of the pairwise areas, and a value of 0 represents none.

Graph-theoretical properties were computed to quantify the topological characteristics of SCNs. For each individual morphological network at each sparsity level, we calculated global metrics including characteristic path length (*L*_*p*_), clustering coefficient (*C*_*p*_), normalized characteristic path length (*λ*), normalized clustering coefficient (*γ*), small-worldness (*σ*), global efficiency (*E*_*glob*_), and local efficiency (*E*_*loc*_). Herein, a set of random networks (number = 1000) were generated for the calculation of *λ* and *γ*. To characterize the network nodes, nodal metrics including nodal degree, nodal efficiency and nodal betweenness, were also computed. We further calculated the area under the curve (AUC) across the sparsity range (0.05–0.30, with an interval of 0.01) [34] as a comprehensive scalar measure of brain network topology for each metric, thereby avoiding potential bias of any single threshold.

### Statistical analyses

Demographic and clinical data were analyzed using the SPSS software (version 27.0.1, IBM Corp., Armonk, NY, USA). For continuous variables, two-sample t-test (evaluating data with normal distribution) or the Mann–Whitney U test (evaluating data not normally distributed) was performed to compare differences between the VM group and HCs. The chi-square test was used to compare differences of categorical variables. The statistical significance threshold was set at *P* < 0.05.

The AUC of network metrics were compared between the VM group and HCs using an independent-sample t-test with sex, age, and education level as covariates of no interest in the GRETNA toolbox. For local topological characteristics, we used the false discovery rate (FDR) to correct for multiple comparisons at a significance level of *P* < 0.05.

The network-based statistics (NBS) method [43], implemented in the GRETNA toolbox, was used to localize specific pairs of regions with altered structural connections in the VM group. First, the edge-by-edge comparison of the strength of edge weight in the SCN matrix was performed between the VM group and HCs using the two-sample t-test. Second, the components that contained connected supra-threshold edges with P value of 0.001 were retained. Then, an empirical distribution of the connected component size was derived, and the largest component size was calculated by repeating the aforementioned steps with 5,000 permutations and setting the P value at 0.05 corrected for multiple comparisons. Before the permutation test, the potential confounding effects of sex, age, and education level were eliminated by multiple linear regression.

In the VM cohort, for network topological properties and connections that exhibited significant between-group differences, partial correlation analyses were performed to examine their relationships with clinical parameters, after controlling for the effects of sex, age, and education level. The correlation analyses were performed with SPSS 27.0.1, and the statistical threshold was set at *P* < 0.05.

### Machine learning analyses

To further validate the between-group differences of network topological properties and connections and to investigate their potential diagnostic value, three classifiers including LR, SVM, and RF were used to construct models for distinguishing individuals with VM from HCs. A combination of significant imaging features was used for the analyses. The nested cross-validation (CV) method was employed for machine learning, with 10-fold CV in the outer loop and stratified 5-fold CV in the inner loop. For the outer loop, min–max normalization was performed in each fold. For the inner loop, hyperparameter tuning was performed to optimize accuracy (LR and SVM: C = 2^− 5^, 2^− 4^, 2^− 3^, 2^− 2^, 2^− 1^, 1, 2, 4, 8, 16, and 32; RF: n_estimators = 10–200 with an interval of 10). After the completion of hyperparameter tuning in the inner loop, the optimal hyperparameters were used to train the final model based on the training set in the outer loop. Subsequently, model validation was performed with the testing set in the outer loop, yielding the accuracy, AUC, sensitivity, and specificity indices. Receiver operating characteristic (ROC) curve analysis was performed to examine classification performance. To validate the significance of accuracy and AUC, nonparametric permutation tests with 5,000 permutations were performed (statistical significance was set at *P* < 0.05). The mean weight (for LR and SVM) as well as the mean feature importance (for RF) across the CVs were employed as indicators of feature contribution to classification. We adopted the top 20% important features (sorted by absolute values of feature contributions) which simultaneously appeared in all models (LR, SVM, and RF) as the most contributed features for distinguishing individuals with VM from HCs.

## Results

### Demographic and clinical characteristics

Table 1 presents the demographic and clinical characteristics of the study population. No significant differences in age (*P* = 0.848), sex (*P* = 0.854), or years of education (*P* = 0.171) were observed between the two groups.

**Table 1:** Demographic and clinical characteristics of VM and HC groups

Items	VM group (*n* = 55)	HC group (*n* = 57)	P
Age (years)	45.89 ± 12.29	45.86 ± 12.37	0.848^a^
Sex (male/female)	8/47	9/48	0.854^b^
Education level (years)	10.47 ± 4.79	11.89 ± 4.77	0.171^a^
Migraine disease duration (years)	11.79 ± 11.21	-	-
Vertigo disease duration (years)	6.95 ± 8.61	-	-
Headache frequency/month	2.32 ± 2.44	-	-
VAS	6.33 ± 2.03	-	-
DHI	52.25 ± 17.04	-	-
MIDAS	11.26 ± 11.77	-	-
HIT-6	55.15 ± 12.73	-	-
PHQ-9	6.36 ± 5.61	-	-
GAD-7	5.04 ± 4.43	-	-

Data were presented as mean ± standard deviation unless otherwise indicated

VM = vestibular migraine; HC = healthy control; VAS = Visual Analog Scale; DHI = Dizziness Handicap Inventory; MIDAS = Migraine Disability Assessment Scale; HIT-6 = Headache Impact Test-6; PHQ-9 = Patient Health Questionnaire-9; GAD-7 = Generalized Anxiety Disorder-7; *n* = number of subjects

^a^ P value with Mann–Whitney U test

^b^ P value with Chi-square test

### Group differences in the topological metrics of SCNs

**Fig. 2:**
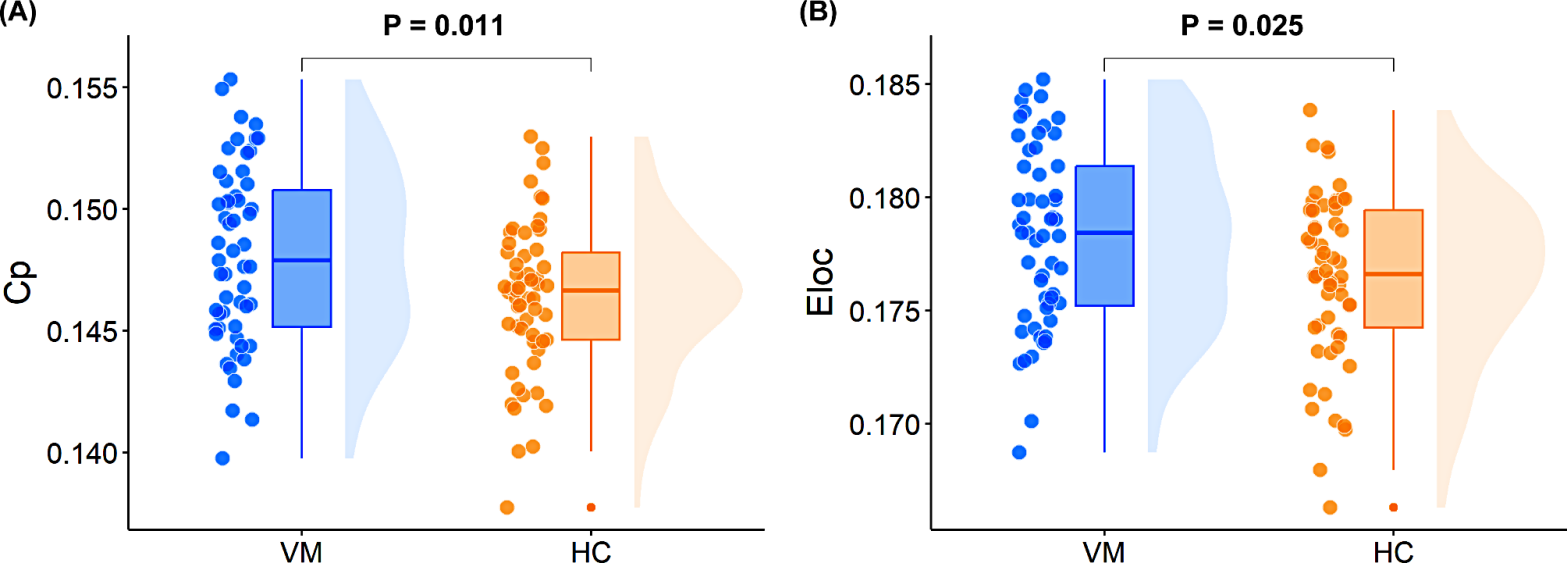
Between-group differences in global topological properties. (**A**) The VM group exhibited significantly increased *C*_*p*_ (*P* = 0.011) compared with HCs. (**B**) The VM group exhibited significantly increased *E*_*loc*_ (*P* = 0.025) compared with HCs. Results are shown as a box plot with individual data points and a smoothed distribution. Sex, age, and education level were controlled as covariates of no interest. VM = vestibular migraine; *C*_*p*_ = clustering coefficient; HC = healthy control; *E*_*loc*_ = local efficiency

Regarding global topological characteristics, patients suffering from VM exhibited significantly increased *C*_*p*_ (*P* = 0.011) and *E*_*loc*_ (*P* = 0.025) compared with HCs, after controlling for sex, age, and education level (Fig. 2). No significant intergroup differences in other global metrics were observed between the two groups. Table 2 presents detailed results of intergroup comparisons of global graph metrics.

**Table 2:** Statistical results of global graph metrics between the two groups (controlling for sex, age, and education level)

Global graph metrics	VM group	HC group	*P*
*L*_*p*_	0.692 ± 0.023	0.691 ± 0.021	0.702
*C*_*p*_	0.148 ± 0.004	0.146 ± 0.003	0.011^*^
*λ*	0.320 ± 0.008	0.317 ± 0.008	0.141
*γ*	0.507 ± 0.053	0.495 ± 0.051	0.414
*σ*	0.391 ± 0.044	0.386 ± 0.042	0.715
*E*_*glob*_	0.098 ± 0.002	0.098 ± 0.002	0.949
*E*_*loc*_	0.178 ± 0.004	0.176 ± 0.004	0.025^*^

Data were presented as mean ± standard deviation

^*^ Statistical significance (*P* < 0.05)

VM = vestibular migraine; HC = healthy control; *L*_*p*_ = characteristic path length; *C*_*p*_ = clustering coefficient; *λ* = normalized characteristic path length; *γ* = normalized clustering coefficient; *σ* = small-worldness; *E*_*glob*_ = global efficiency; *E*_*loc*_ = local efficiency

**Fig. 3:**
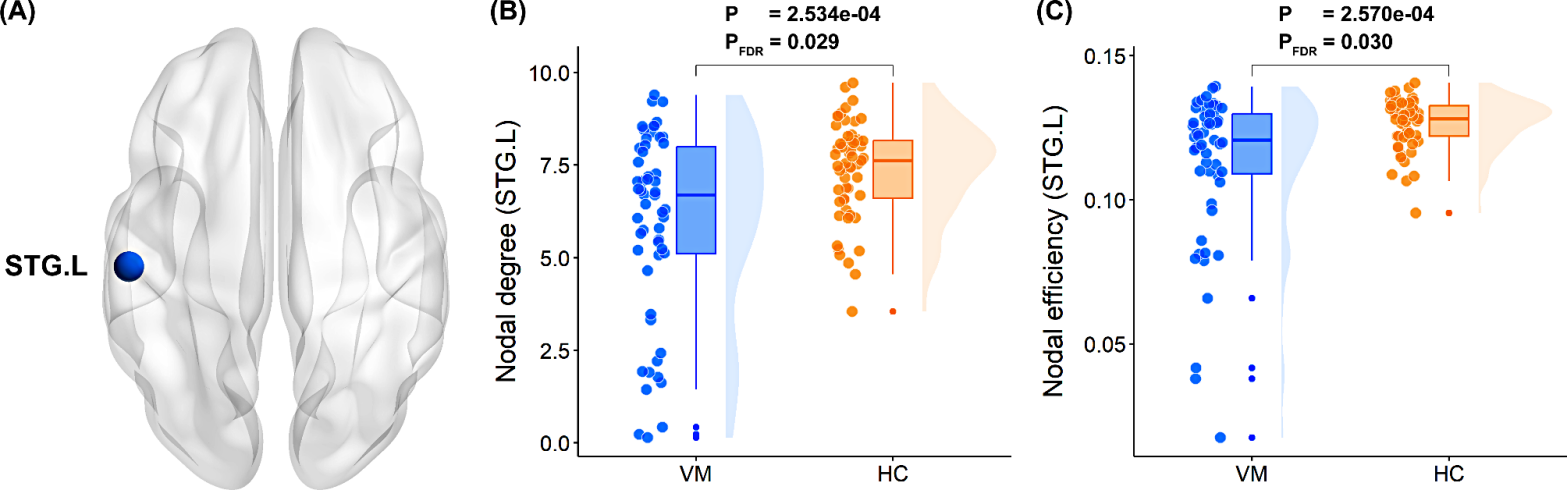
Between-group differences in local topological properties. (**A**) The brain region with significant local graph metrics (i.e., STG.L) was visualized using the BrainNet viewer package (http://nitrc.org/projects/bnv/). (**B**) The VM group demonstrated significantly reduced nodal degree (*P* = 2.534e-04, P_FDR_ = 0.029) in the STG.L compared with HCs. (**C**) The VM group exhibited significantly reduced nodal efficiency (*P* = 2.570e-04, P_FDR_ = 0.030) in the STG.L compared with HCs. Results are shown as a box plot with individual data points and a smoothed distribution. Sex, age, and education level were controlled as covariates of no interest. STG = superior temporal gyrus; L = left; VM = vestibular migraine; FDR = false discovery rate; HC = healthy control

Regarding local topological characteristics, patients suffering from VM exhibited significantly reduced nodal degree (*P* = 2.534e-04, FDR-corrected P [P_FDR_] = 0.029) and nodal efficiency (*P* = 2.570e-04, P_FDR_ = 0.030) in the left superior temporal gyrus (STG) after controlling for sex, age, and education level (Fig. 3; Table 3). No significant intergroup differences were observed in the other local metrics.

**Table 3:** Statistical results of significant local graph metrics between the two groups (controlling for sex, age, and education level)

Local graph metrics	VM group	HC group	P	P_FDR_
Nodal degree (STG.L)	5.91 ± 2.55	7.38 ± 1.31	2.534e-04	0.029^*^
Nodal efficiency (STG.L)	0.11 ± 0.03	0.13 ± 0.01	2.570e-04	0.030^*^

Data were presented as mean ± standard deviation

^*^ Statistical significance (P_FDR_ < 0.05)

VM = vestibular migraine; HC = healthy control; FDR = false discovery rate; STG = superior temporal gyrus; L = left

### Group differences in network connections

**Fig. 4:**
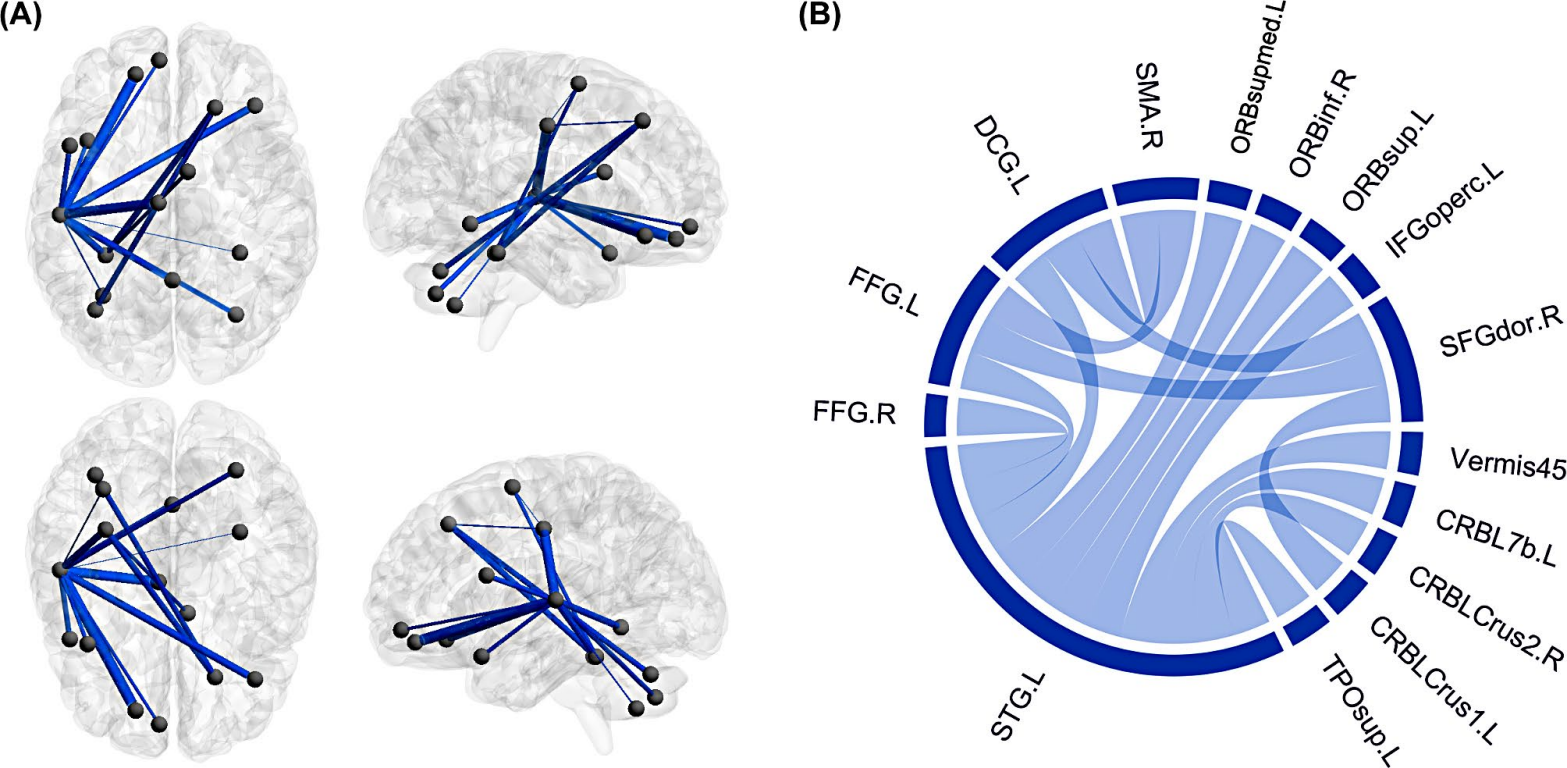
Illustrations of the impaired interregional morphological connectivity in VM. (**A**) The identified connected component with decreased connectivity was mapped on the Ch2 template using the BrainNet viewer package (http://nitrc.org/projects/bnv/). Blue line indicates the weight of the decreased connection in the VM group. (**B**) The connectogram presents detailed information of the nodes and edges in the component. Blue chord indicates the existence of reduced interregional connection in the VM group. Sex, age, and education level were controlled as covariates of no interest. VM = vestibular migraine; SFGdor = superior frontal gyrus, dorsolateral; IFGoperc = inferior frontal gyrus, opercular part; ORBsup = superior frontal gyrus, orbital part; ORBinf = inferior frontal gyrus, orbital part; ORBsupmed = superior frontal gyrus, medial orbital; SMA = supplementary motor area; DCG = median cingulate and paracingulate gyri; FFG = fusiform gyrus; STG = superior temporal gyrus; TPOsup = temporal pole: superior temporal gyrus; CRBLCrus1 = crus I of cerebellar hemisphere; CRBLCrus2 = crus II of cerebellar hemisphere; CRBL7b = lobule VIIB of cerebellar hemisphere; Vermis45 = lobule IV, V of vermis; L = left; R = right

Compared with HCs, the VM group presented one connected component with decreased morphological connectivity strength in the NBS analysis, after controlling for sex, age, and education level (*P* < 0.05, NBS corrected). The component comprised 15 brain regions and 16 edges. The involved regions were mainly located in the STG, temporal pole, prefrontal cortex (PFC), supplementary motor area (SMA), cingulum, fusiform gyrus, and cerebellum (Fig. 4).

### Correlation analysis

**Fig. 5:**
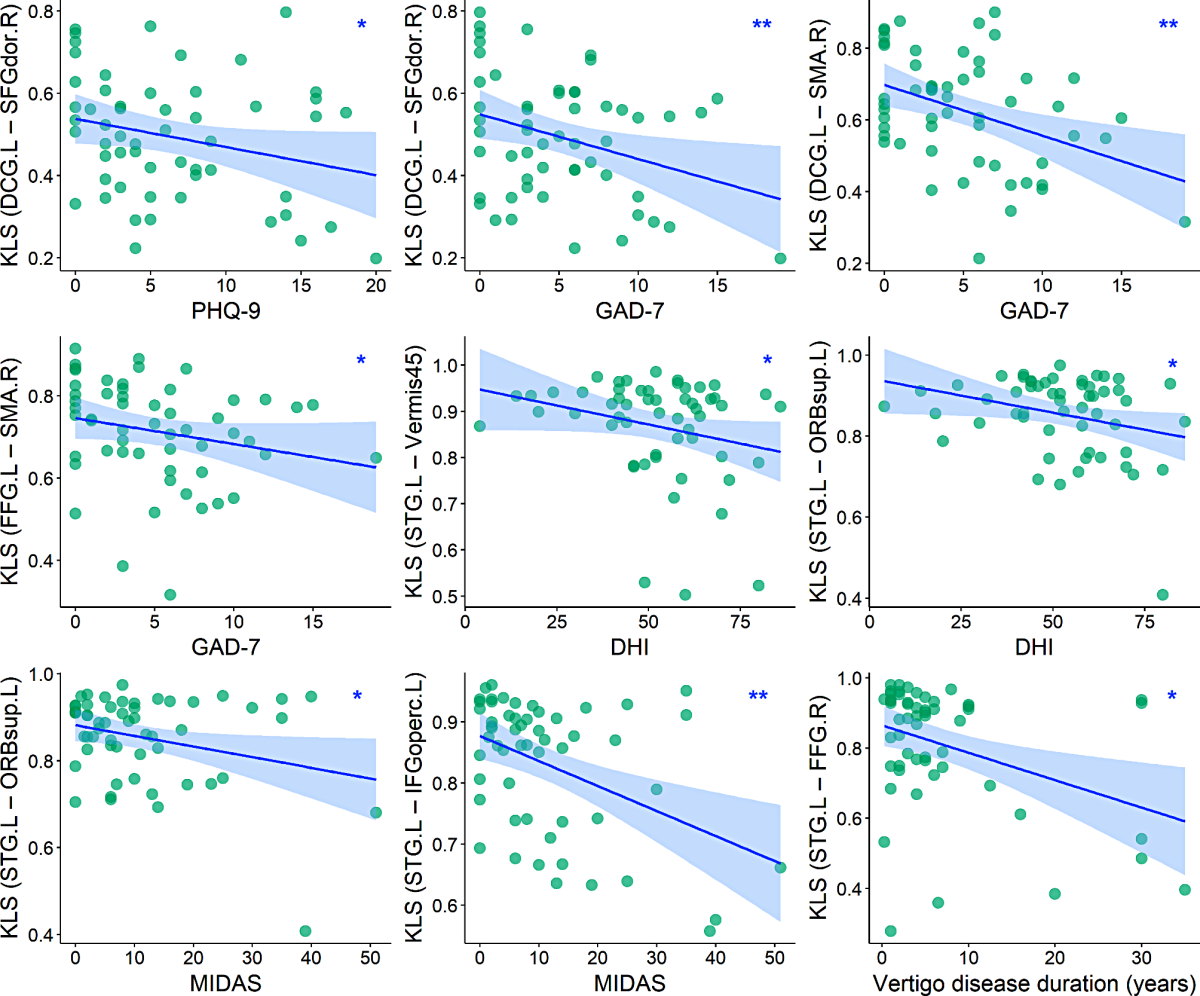
Clinical correlates of significant GM connectome features in the VM group. Scatter plots depict the relationship between clinical indices and GM connectome features with between-group differences in patients with VM (^∗^*P* < 0.05, ^∗∗^*P* < 0.01). Several connections in the identified connected component were correlated with clinical measures (*P* < 0.05); however, these correlations did not survive FDR correction (P_FDR_ > 0.05) or Bonferroni correction (Bonferroni-corrected *P* > 0.05) for multiple comparisons. Sex, age, and education level were controlled as covariates of no interest. GM = gray matter; VM = vestibular migraine; FDR = false discovery rate; KLS = Kullback–Leibler divergence-based similarity; DCG = median cingulate and paracingulate gyri; SFGdor = superior frontal gyrus, dorsolateral; SMA = supplementary motor area; FFG = fusiform gyrus; STG = superior temporal gyrus; Vermis45 = lobule IV, V of vermis; ORBsup = superior frontal gyrus, orbital part; IFGoperc = inferior frontal gyrus, opercular part; L = left; R = right; PHQ-9 = Patient Health Questionnaire-9; GAD-7 = Generalized Anxiety Disorder-7; DHI = Dizziness Handicap Inventory; MIDAS = Migraine Disability Assessment Scale

In the VM group, after controlling for sex, age, and education level, several connections in the identified connected component were correlated with clinical measures, including vertigo disease duration, DHI, MIDAS, PHQ-9, and GAD-7 (*P* < 0.05) (Fig. 5). However, these correlations did not survive FDR correction (P_FDR_ > 0.05) or Bonferroni correction (Bonferroni-corrected *P* > 0.05) for multiple comparisons. Furthermore, no significant relationships were observed between the other network features and clinical indices (*P* > 0.05).

### Single‑subject classification of patients with VM and HCs

**Fig. 6:**
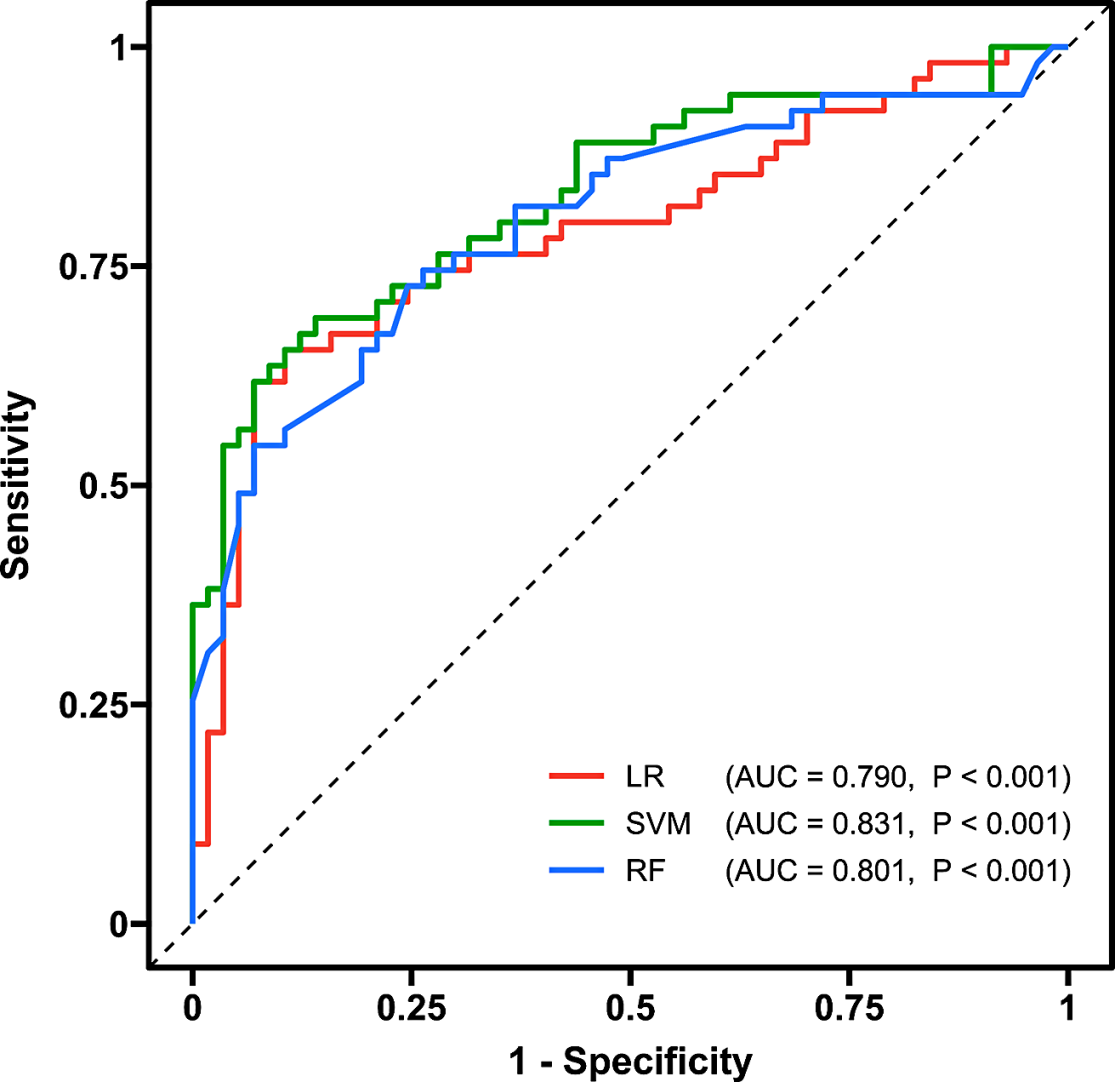
ROC curves of the LR, SVM, and RF classifiers based on discriminative GM connectome features (i.e., significant topological metrics and connections) for distinguishing individuals with VM from HCs. The AUCs for the LR, SVM and RF models were 0.790 (*P* < 0.001), 0.831 (*P* < 0.001) and 0.801 (*P* < 0.001), respectively. ROC = receiver operating characteristic; LR = logistic regression; SVM = support vector machine; RF = random forest; GM = gray matter; VM = vestibular migraine; HC = healthy control; AUC = area under the curve

Based on the significant topological metrics and connections, our machine learning models exhibited good performance in distinguishing patients with VM from HCs. Specifically, the LR model achieved a total accuracy of 77.68% (*P* < 0.001), an AUC of 0.790 (*P* < 0.001), a sensitivity of 65.45%, and a specificity of 89.47%; the SVM model achieved a total accuracy of 77.68% (*P* < 0.001), an AUC of 0.831 (*P* < 0.001), a sensitivity of 63.64%, and a specificity of 91.23%; and the RF model achieved a total accuracy of 72.32% (*P* < 0.001), an AUC of 0.801 (*P* < 0.001), a sensitivity of 67.27%, and a specificity of 77.19%. The ROC curves of the classifiers are shown in Fig. 6. The most contributed features (i.e., the top 20% important features simultaneously appearing in all models) were the KLS between left fusiform gyrus and right superior frontal gyrus (dorsolateral), as well as the KLS between left crus I of cerebellar hemisphere and right superior frontal gyrus (dorsolateral). Detailed contributions of features in each model are shown in Table S2.

## Discussion

This study investigated single-subject GM connectome disorganization in patients with VM using a novel morphological similarity network analysis combined with a machine learning approach. Consistent with our hypothesis, the VM group exhibited altered global and regional topological properties, and reduced connectivity in a specific component. Moreover, the machine learning models achieved good accuracy and efficacy in classifying patients with VM and HCs. These findings may provide new insights into the pathophysiology of VM.

The morphological similarity network has been considered as a promising approach for investigating the individual-level structural organization of the human brain [41]. Although the exact interpretation of neurobiological meaning remains unclear, several pieces of evidence have shown that heredity, experience-related plasticity, development, and aging trajectories play crucial roles in the formation of the network [30, 44]. Physically, the cortical morphology of the human brain comprises a complex but efficient network that balances local specialization and global integration to maximize parallel information processing [41]. Under pathological conditions, the induced changes in GM density may influence the morphological similarity of different GM regions, thus disrupting the distributed GM morphological network architecture [32]. Therefore, the morphological similarity network is thought to be biologically meaningful for capturing the potential mechanisms underlying these physical and pathological processes [41].

The normal human brain is globally organized as a small-world network, which optimally balances segregation (reflected by *C*_*p*_, *γ*, and *E*_*loc*_) and integration (reflected by *L*_*p*_, *λ*, and *E*_*glob*_) to facilitate efficient information processing at minimal costs [45]. In disease states, small-world topological properties can be altered and categorized into four patterns: regularization, randomization, stronger small-worldization, and weaker small-worldization [46]. Our study demonstrated that despite exhibiting an overall small-world architecture similar to that of HCs, the VM group exhibited higher *C*_*p*_ and *E*_*loc*_, indicating higher segregation of the structural network and suggesting a regularization profile in patients with VM. This finding agrees with those of a previous investigation by Liu et al. [47], which revealed a similar regularization (higher *C*_*p*_ and *γ*) of structural network topology in patients with migraine. They explained that the presence of chronic headache alters the structural connections sharing the nested local network in a chronic pain-related manner, inducing a more clustered condition. Thus, the observed regularization pattern may be partially attributed to the outcomes of headache in VM pathophysiology.

At the regional level, decreased nodal degree and efficiency in the left STG were observed in the VM group. Nodal degree and efficiency reflect the capacity of information integration and transmission, whereas a reduction in these parameters indicates disrupted interconnectivity with other regions in the network [48]. GM volume reduction [20] and various functional changes [12, 49, 50] of the STG in patients with VM have been reported, suggesting that the STG plays an important role in the neural mechanism underlying VM. The STG, a crucial area within the vestibular network [51], participates in processing the spatial coordination of the eyes, head, and body [52], and is closely connected to the multisensory parieto-insula cortex [20]. Furthermore, the STG is implicated in emotional perception [53]. Combined with the clinical manifestations of VM (i.e., dizziness, pain, and emotional discomfort), we deduced that the observed reduced nodal properties of the STG may reflect potential disturbances in the ability to process information related to spatial coordination, multisensory integration, and emotional perception. However, it should be noted that a portion of values (including several outliers) deviated from the overall pattern for both nodal degree and nodal efficiency in the left STG of VM, thereby exerting an influence on the general level of these indices in the VM cohort. We cautiously hypothesized that this finding might reflect the individual heterogeneity of VM, given that VM is recognized to be a highly heterogeneous disorder with variations in clinical presentations [54]. Nonetheless, the hypothesis was speculative and needed to be validated by further research with a larger cohort.

Our examination of the morphological connections between brain region pairs using NBS identified a subnetwork with decreased connections in patients with VM, mainly involving the STG, temporal pole, PFC, SMA, cingulum cortex, fusiform gyrus, and cerebellum. Considering the aforementioned pivotal role of the STG in the pathophysiology of VM, it would be reasonable that the STG appeared in the disrupted component. The temporal pole is an associative multisensory region that processes visual, olfactory, and auditory information [55]. Considering that some patients with VM exhibit enhanced sensitivity to olfactory, auditory, and visual stimuli [56, 57], we assume that temporal pole alterations might be related to the hypersensitivity manifestations of VM. The PFC plays a crucial role in connecting the limbic system and subcortical regions, and is implicated in pain perception, regulation, as well as cognitive and emotional processes [58, 59]. A previous study revealed that migraineurs exhibited greater pain-induced activation in the PFC, suggesting a link to the cognitive aspects of pain perception, such as pain-related memories [60]. Therefore, PFC involvement may be associated with painful experiences in individuals with VM.

The SMA, located in the dorsomedial frontal cortex, is involved in self-initiated and triggered movements [61], as well as anticipation and affective components of pain [62]. The observed SMA involvement is consistent with previous neuroimaging investigations on VM [19, 62], suggesting deficits in pain and balance control, which may contribute to hyperreactivity, equilibrium problems, and motion-induced vestibular symptoms in patients with VM. The cingulate cortex plays a vital role not only in pain sensation [63], but also in motor and vestibular processing [64]; thus, it is plausible that the cingulate cortex was involved. The fusiform gyrus is implicated in higher visual function and pain processing [65, 66] and has been reported to exhibit structural and functional disturbances in migraineurs [60, 65]. The cerebellum plays a key role in processing sensorimotor, cognitive, and emotional information [67]. Structural abnormalities in the cerebellum may reflect both the physical (i.e., dizziness, pain) and mental (i.e., anxiety, depression [68]) aspects of the disease. Taken together, we show that patients with VM exhibited altered morphological relations between these regions, indicating an incongruous GM disruption pattern, which may be a possible neural mechanism underlying VM. Furthermore, in this study, several altered connections tended to be correlated with clinical indices (i.e., vertigo disease duration, DHI, MIDAS, PHQ-9, and GAD-7), indicating that these observed changes may be meaningful neuroimaging features for VM.

In our study, we identified two morphological connections as the most contributed GM connectome features, including the KLS between fusiform gyrus and PFC, as well as the KLS between cerebellum and PFC. The cerebellum and PFC are vital components of the vestibular-thalamic-cortical pathway, whilst the fusiform gyrus is located in the ventral visual stream. In a previous FDG-PET study by Shin et al. [69], patients with VM showed activation in the vestibular-thalamic-cortical pathway and inhibition in the visual pathway during the ictal period. Another fMRI investigation [50] on the vertigo-free period demonstrated activation of brain regions associated with integration of visual and vestibular cues. Consistent with these findings, the present study also revealed changes within these circuits. Although the neurobiological meaning remains incompletely understood, morphological similarity is deemed to contain specific information (e.g. cytoarchitectonic similarity and co-expression of specialized neuronal function genes), and can be driven by other factors such as levels of neurophysiological activity [32, 70]. While in pathological states, lesions may induce GM density changes which impact the inter-regional morphological similarities [32]. Thus, we deduce that the altered morphological relations might indicate the distinct integrity of different portions in the relevant circuits [71], hinting that disruption of the components in the circuits may be incongruous. As a supplement to prior research, the findings elucidated the potential morphological relation mechanisms regarding the pathways involved in VM.

Currently, most neuroimaging studies draw conclusions based on differences identified through traditional intergroup statistical approaches. However, these findings cannot be further applied at the individual level, limiting their translational capability. Underdiagnosis of VM is frequently observed in clinical practice [72], likely due to the unclear pathophysiological mechanisms and the lack of specific biomarkers [73]. In this study, we used three common machine learning models (LR, SVM, and RF) based on the topological metrics and connections of a morphological similarity network. The individualized SCN resolved the limitation of conventional group-level SCN (i.e., inability to generate an individual network for each subject), and the derived discriminative features achieved good performance in distinguishing individuals suffering from VM from HCs and may thus serve as objective and effective biomarkers of VM. However, despite the potential value demonstrated by our findings, it should be noted that this study is preliminary in nature, and further research is required to validate the generalizability of the present results.

Our study has several limitations. First, the sample size was only relatively larger than those of the existing research. Further studies with more participants would be helpful to verify the present results. Second, the subjects were recruited from a single center, and the robustness of the proposed model and validity of the results require further validation and optimization in a multicenter dataset. Finally, the cross-sectional study design limited us from inferring the causal relationship between abnormalities in the morphological similarity network and VM attacks.

## Conclusion

Based on single-subject GM covariance connectome analyses, this study revealed that patients with VM had altered global and regional topological properties, as well as reduced network connections in a specific component, reflecting potential dizziness, pain, and emotional dysfunctions. These findings would enhance our current understanding of VM from the viewpoint of morphological similarity network. The identified discriminative SCN features could serve as individualized neuroimaging markers of VM.

## Electronic supplementary material

Below is the link to the electronic supplementary material.

Supplementary Material 1

## Data Availability

The datasets used and/or analysed during the current study are available from the corresponding author on reasonable request.

## Abbreviations

AAL: Automatic Anatomical Labeling
AUC: Area under the curve
CAT: Computational Anatomy Toolbox
C_p_: Clustering coefficient
CRBL7b: Lobule VIIB of cerebellar hemisphere
CRBLCrus1: Crus I of cerebellar hemisphere
CRBLCrus2: Crus II of cerebellar hemisphere
CV: Cross-validation
DCG: Median cingulate and paracingulate gyri
DHI: Dizziness Handicap Inventory
E_glob_: Global efficiency
E_loc_: Local efficiency
FDR: False discovery rate
FFG: Fusiform gyrus
GAD-7: Generalized Anxiety Disorder-7
GM: Gray matter
HC: Healthy control
HIT-6: Headache Impact Test-6
IFGoperc: Inferior frontal gyrus, opercular part
KL: Kullback–Leibler
KLS: KL divergence-based similarity
L: Left
L_p_: Characteristic path length
LR: Logistic regression
MIDAS: Migraine Disability Assessment Scale
n: Number of subjects
NBS: Network-based statistics
ORBinf: Inferior frontal gyrus, orbital part
ORBsup: Superior frontal gyrus, orbital part
ORBsupmed: Superior frontal gyrus, medial orbital
PDF: Probability Distribution Function
PFC: Prefrontal cortex
PHQ-9: Patient Health Questionnaire-9
R: Right
RF: Random forest
ROC: Receiver operating characteristic
ROI: Region of interest
rs-fMRI: Resting-state functional MRI
SCN: Structural covariance network
SFGdor: Superior frontal gyrus, dorsolateral
SMA: Supplementary motor area
sMRI: Structural MRI
SPM: Statistical Parametric Mapping
STG: Superior temporal gyrus
SVM: Support vector machine
TPOsup: Temporal pole: superior temporal gyrus
VAS: Visual Analog Scale
VBM: Voxel-based morphometry
Vermis45: Lobule IV, V of vermis
VM: Vestibular migraine
γ: Normalized clustering coefficient
λ: Normalized characteristic path length
σ: Small-worldness
