# Supplementary material for: Disrupted gray matter connectome in vestibular migraine: a combined machine learning and individual-level morphological brain network analysis
Source: J Headache Pain. 2024 Oct 11;25(1):177. doi: 10.1186/s10194-024-01861-9 (PMC11468853; doi:10.1186/s10194-024-01861-9)
Supplement: Supplementary file 1 — Supplementary Material 1 [file 10194_2024_1861_MOESM1_ESM.doc]

**Supplementary Material**

**Table S1.** Neuroimaging studies on structural brain changes in patients with VM

| **References** | **Methodology** | **Sample size** | **Minimum *t*** | **Effect size** |
| --- | --- | --- | --- | --- |
| Obermann et al., 2014 | VBM | 17 VM, 17 HC | 3.40 | 1.20 |
| Messina et al., 2017 | VBM | 19 VM, 20 HC | 3.10 | 1.02 |
| Zhe et al., 2020 | VBM | 20 VM, 20 HC | 4.39 | 1.42 |
| Zhe et al., 2021 | VBM | 30 VM, 30 HC | 5.60 | 1.47 |
| Zhe et al., 2021 | SBM | 25 VM, 27 HC | 4.91 | 1.39 |

The effect size in these prior studies was described using Cohen’s d that was calculated based on the equation (Cohen, 1992):


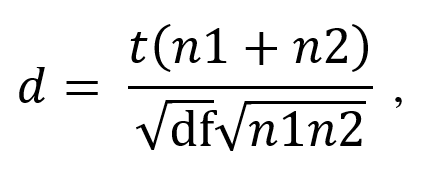


where *t* and df are the minimum *t* value and the degree of freedom in the comparison analysis, whilst *n*1 and *n*2 are the sample sizes of patients and controls.

VM = vestibular migraine; HC = healthy control; VBM = voxel-based morphometry; SBM = surface-based morphometry

**Table S2.** Contribution of significant GM connectome features to classification in each machine learning model

| **Features** | **Contribution** **to classification** | | |
| --- | --- | --- | --- |
|  | **LR** | **SVM** | **RF** |
| *C_p_* | 0.419 | 0.505 | 0.047 |
| *E_loc_* | 0.339 | 0.400 | 0.047 |
| Nodal degree (STG.L) | -0.096 | -0.241 | 0.043 |
| Nodal efficiency (STG.L) | -0.150 | -0.170 | 0.035 |
| KLS (DCG.L – SFGdor.R) | -0.435 | -0.707 | 0.058 |
| KLS (DCG.L – SMA.R) | -0.234 | -0.312 | 0.037 |
| KLS (FFG.L – SFGdor.R) | -0.792 | -0.975 | 0.081 |
| KLS (FFG.L – SMA.R) | -0.540 | -0.529 | 0.058 |
| KLS (STG.L – ORBsup.L) | -0.260 | -0.215 | 0.036 |
| KLS (STG.L – IFGoperc.L) | -0.795 | -0.694 | 0.056 |
| KLS (STG.L – ORBinf.R) | -0.064 | -0.284 | 0.055 |
| KLS (STG.L – ORBsupmed.L) | -0.586 | -0.644 | 0.043 |
| KLS (STG.L – DCG.L) | -0.466 | -0.373 | 0.054 |
| KLS (STG.L – FFG.L) | -0.128 | -0.296 | 0.029 |
| KLS (STG.L – FFG.R) | -0.312 | -0.246 | 0.041 |
| KLS (STG.L – TPOsup.L) | -1.076 | -0.514 | 0.051 |
| KLS (STG.L – CRBLCrus2.R) | -0.188 | -0.270 | 0.037 |
| KLS (STG.L – CRBL7b.L) | -0.257 | -0.064 | 0.049 |
| KLS (STG.L – Vermis45) | -0.631 | -0.446 | 0.067 |
| KLS (CRBLCrus1.L – SFGdor.R) | -0.764 | -1.060 | 0.075 |

The mean weight (for LR and SVM) as well as the mean feature importance (for RF) across the CVs were reported as feature contribution indicators.

GM = gray matter; LR = logistic regression; SVM = support vector machine; RF = random forest; *C_p_* = clustering coefficient; *E_loc_* = local efficiency; STG = superior temporal gyrus; KLS = Kullback–Leibler divergence-based similarity; DCG = median cingulate and paracingulate gyri; SFGdor = superior frontal gyrus, dorsolateral; SMA = supplementary motor area; FFG = fusiform gyrus; ORBsup = superior frontal gyrus, orbital part; IFGoperc = inferior frontal gyrus, opercular part; ORBinf = inferior frontal gyrus, orbital part; ORBsupmed = superior frontal gyrus, medial orbital; TPOsup = temporal pole: superior temporal gyrus; CRBLCrus2 = crus II of cerebellar hemisphere; CRBL7b = lobule VIIB of cerebellar hemisphere; Vermis45 = lobule IV, V of vermis; CRBLCrus1 = crus I of cerebellar hemisphere; L = left; R = right; CV = cross-validation

**References**

1. Obermann M, Wurthmann S, Steinberg BS, Theysohn N, Diener HC, Naegel S (2014). Central vestibular system modulation in vestibular migraine. Cephalalgia, 34(13):1053-61.

2. Messina R, Rocca MA, Colombo B, et al. (2017). Structural brain abnormalities in patients with vestibular migraine. J Neurol, 264(2):295-303.

3. Zhe X, Gao J, Chen L, et al. (2020). Altered structure of the vestibular cortex in patients with vestibular migraine. Brain Behav, 10(4):e01572.

4. Zhe X, Zhang X, Chen L, et al. (2021). Altered Gray Matter Volume and Functional Connectivity in Patients With Vestibular Migraine. Front Neurosci, 15:683802.

5. Zhe X, Chen L, Zhang D, et al. (2021). Cortical Areas Associated With Multisensory Integration Showing Altered Morphology and Functional Connectivity in Relation to Reduced Life Quality in Vestibular Migraine. Front Hum Neurosci, 15:717130.

6. Cohen J (1992). A power primer. Psychol Bull, 112(1):155-9.
